# Tropism of Newcastle disease virus strains for chicken neurons, astrocytes, oligodendrocytes, and microglia

**DOI:** 10.1186/s12917-019-2053-z

**Published:** 2019-09-04

**Authors:** Salman L. Butt, Veridiana Maria Brianezi Dignani Moura, Leonardo Susta, Patti J. Miller, Jessica M. Hutcheson, Stivalis Cardenas-Garcia, Corrie C. Brown, Franklin D. West, Claudio L. Afonso, James B. Stanton

**Affiliations:** 1 Department of Pathology, College of Veterinary Medicine, University of Georgia, Athens, GA USA; 2 Southeast Poultry Research Laboratory, Agricultural Research Service, USDA, Athens, GA USA; 3 Animal Pathology, School of Veterinary Medicine and Animal Science, Federal University of Goiás, Goiânia, GO Brazil; 4 Department of Pathobiology, Ontario Veterinary College, University of Guelph, Guelph, Ontario N1G 2W1 Canada; 5 Department of Population Health, College of Veterinary Medicine, Athens, GA USA; 6 Regenerative Bioscience Center, University of Georgia, Athens, GA USA; 7 Department of Animal and Dairy Science, College of Agricultural and Environmental Sciences, University of Georgia, Athens, GA USA

**Keywords:** Newcastle disease virus, Primary chicken neural cells, Double immunofluorescence, Tropism, Neurotropism, Paramyxovirus

## Abstract

**Background:**

Newcastle disease (ND), which is caused by infections of poultry species with virulent strains of Avian orthoavulavirus-1, also known as avian paramyxovirus 1 (APMV-1), and formerly known as Newcastle disease virus (NDV), may cause neurological signs and encephalitis. Neurological signs are often the only clinical signs observed in birds infected with neurotropic strains of NDV. Experimental infections have shown that the replication of virulent NDV (vNDV) strains is in the brain parenchyma and is possibly confined to neurons and ependymal cells. However, little information is available on the ability of vNDV strains to infect subset of glial cells (astrocytes, oligodendrocytes, and microglia). The objective of this study was to evaluate the ability of NDV strains of different levels of virulence to infect a subset of glial cells both in vitro and in vivo. Thus, neurons, astrocytes and oligodendrocytes from the brains of day-old White Leghorn chickens were harvested, cultured, and infected with both non-virulent (LaSota) and virulent, neurotropic (TxGB) NDV strains. To confirm these findings in vivo, the tropism of three vNDV strains with varying pathotypes (SA60 [viscerotropic], TxGB [neurotropic], and Tx450 [mesogenic]) was assessed in archived formalin-fixed material from day-old chicks inoculated intracerebrally.

**Results:**

Double immunofluorescence for NDV nucleoprotein and cellular markers showed that both strains infected at least 20% of each of the cell types (neurons, astrocytes, and oligodendrocytes). At 24 h post-inoculation, TxGB replicated significantly more than LaSota. Double immunofluorescence (DIFA) with markers for neurons, astrocytes, microglia, and NDV nucleoprotein detected the three strains in all three cell types at similar levels.

**Conclusion:**

These data indicate that similar to other paramyxoviruses, neurons and glial cells (astrocytes, oligodendrocytes, and microglia) are susceptible to vNDV infection, and suggest that factors other than cellular tropism are likely the major determinant of the neurotropic phenotype.

**Electronic supplementary material:**

The online version of this article (10.1186/s12917-019-2053-z) contains supplementary material, which is available to authorized users.

## Background

Newcastle disease (ND), which is caused by virulent strains of avian orthoavulavirus 1 (formerly designated as Avian avulavirus 1, commonly known as avian paramyxovirus 1, or Newcastle disease virus, NDV, used in this paper) [1], is an important, worldwide poultry disease responsible for major panzootics and extensive poultry mortality [2]. The virus is classified in the order *Mononegavirales*, family *Paramyxoviridae*, subfamily *Avulavirinae*, and genus *Orthoavulavirus* [3]. Based upon the clinical signs induced in naive chickens, NDV strains have been classified into four pathotypes, from the least to the most virulent: asymptomatic enteric, lentogenic, mesogenic, and velogenic [4]. Mesogenic and velogenic strains are defined as virulent. Velogenic strains can be further divided into velogenic viscerotropic (VVNDV), which cause acute, hemorrhagic lesions throughout the gastrointestinal tract and high mortality, and velogenic neurotropic (VNNDV), which cause significant neurologic signs and high mortality (typically lower mortality than VVNDV) [2, 4]. The intracerebral pathogenicity index (ICPI), is the World Animal Health Organization protocol for defining NDV pathogenicity [5], consists of inoculating virus into the cerebrum of one-day-old SPF (or NDV-antibody free) chickens and deriving a clinical weighted score that ranges from 0.0 to 2.0 [6]. Scores ≥0.7 classify a strain as virulent (vNDV) [5]. Typically, ICPI values for mesogenic strains are from 0.7 to 1.5, and from 1.5 to 2.0 for velogenic strains [6].

The main molecular determinant of NDV virulence is the fusion protein (F) cleavage site [7]. Virulent NDV strains have at least three multiple basic amino acid residues between position 113 and 116 with a phenylalanine at position 117, which allows for cleavage via ubiquitous host proteases and ultimately systemic spread. In contrast, non-virulent strains lack the polybasic configuration phenylalanine combination and cleavage is dependent on trypsin-like proteases [8], which are mainly found in the intestinal and respiratory tract, leading to localized infection in these systems [7].

Virulent NDV strains can replicate in the central nervous system (CNS), causing various degrees of non-suppurative encephalitis, and severe neurological signs [4, 9]. In several animal experiments conducted by our group, 4-week-old specific pathogen free (SPF) chickens infected with vNDV strains through eyelid instillation, viral antigen in the CNS (as assessed by immunohistochemistry [IHC]) was in neurons scattered throughout the brain, without an obvious predilection for specific brain regions [9–11]. A retrospective review of archived material from these studies, specifically focusing on CNS histopathology, showed that NDV immunoreactivity was detected mainly in cells morphologically consistent with neurons and infrequently in undefined glial cells [9]. In a study detailing the timing of nervous infection by NDV following intranasal and conjunctival inoculation of 3-week-old chicks, NDV antigen was detected in neurons, endothelium, and undefined glial cells and replication of NDV LaSota strain was restricted to ependymal cells of brain tissue [12]. In the mentioned studies, NDV-positive cells were not identified by specific immunolabeling. In a recent study conducted by intracerebral inoculation of vNDV strains (velogenic and mesogenic) into day-old SPF chickens, viral antigen (assessed by single staining IHC) was observed predominantly in cells morphologically consistent with neurons, ependymal cells, and less frequently in astrocyte-like cells, especially in those areas where extensive virus damage was observed [13]. Therefore, based on the data reported in the literature, it is likely that NDV infects glial cells. However, the extent of NDV infection of glial cells and the types of affected glial cells remain unknown. Furthermore, it is unclear if the differential ability of certain NDV strains to replicate in the cellular subsets of the neuroparenchyma (e.g., neurons, astrocytes and oligodendrocytes) contributes to neuroinvasion and overall neuropathogenesis in chickens. Virus replication in glial cells is an important pathogenetic mechanism for other members of the *Paramyxoviridae* family, such as *Morbilliviruses* (i.e., distemper and measles viruses), which extensively replicate in astrocytes, microglia and, to lesser extent, oligodendrocytes. This has been demonstrated to be important for the demyelination that is typical of paramyxoviral encephalitides [14, 15]. Further, the ability to replicate in glial cells could influence NDV neuroinvasion, as astrocytes are the main component of the *glia limitans*, which contributes to separation of the brain parenchyma from the blood and the cerebrospinal fluid (CSF), both possible portals of entry for pathogens into the CNS [16].

In order to assess the ability of NDV strains to differentially infect and replicate in neurons, astrocytes and oligodendrocytes in vitro, primary mixed chicken neural cell cultures were obtained from SPF embryos and infected with one virulent and one non-virulent NDV strain. Permissibility of these mixed neural cells to infection was assessed by measuring viral titers produced at 24 h post-inoculation. Infection of cell types was assayed by double immunofluorescence assay (DIFA) for NDV and cellular markers (neurons, astrocytes, and oligodendrocytes). Additionally, DIFA (NDV and neurons, astrocytes, or microglia) was conducted to confirm tropism on archived brain tissue harvested from day-old chickens inoculated intracerebrally with three virulent strains with varying virulence (SA60 [viscerotropic], TxGB [neurotropic], and Tx450 [mesogenic]) [13]. Double immunofluorescence assay was carried out using markers for neurons, astrocytes, microglia, and NDV nucleoprotein. This is the first study to utilize primary chicken neural cells and DIFA to investigate NDV neuropathogenesis.

## Results

### Primary culture and characterization of chicken neural cells

**Fig. 1:**
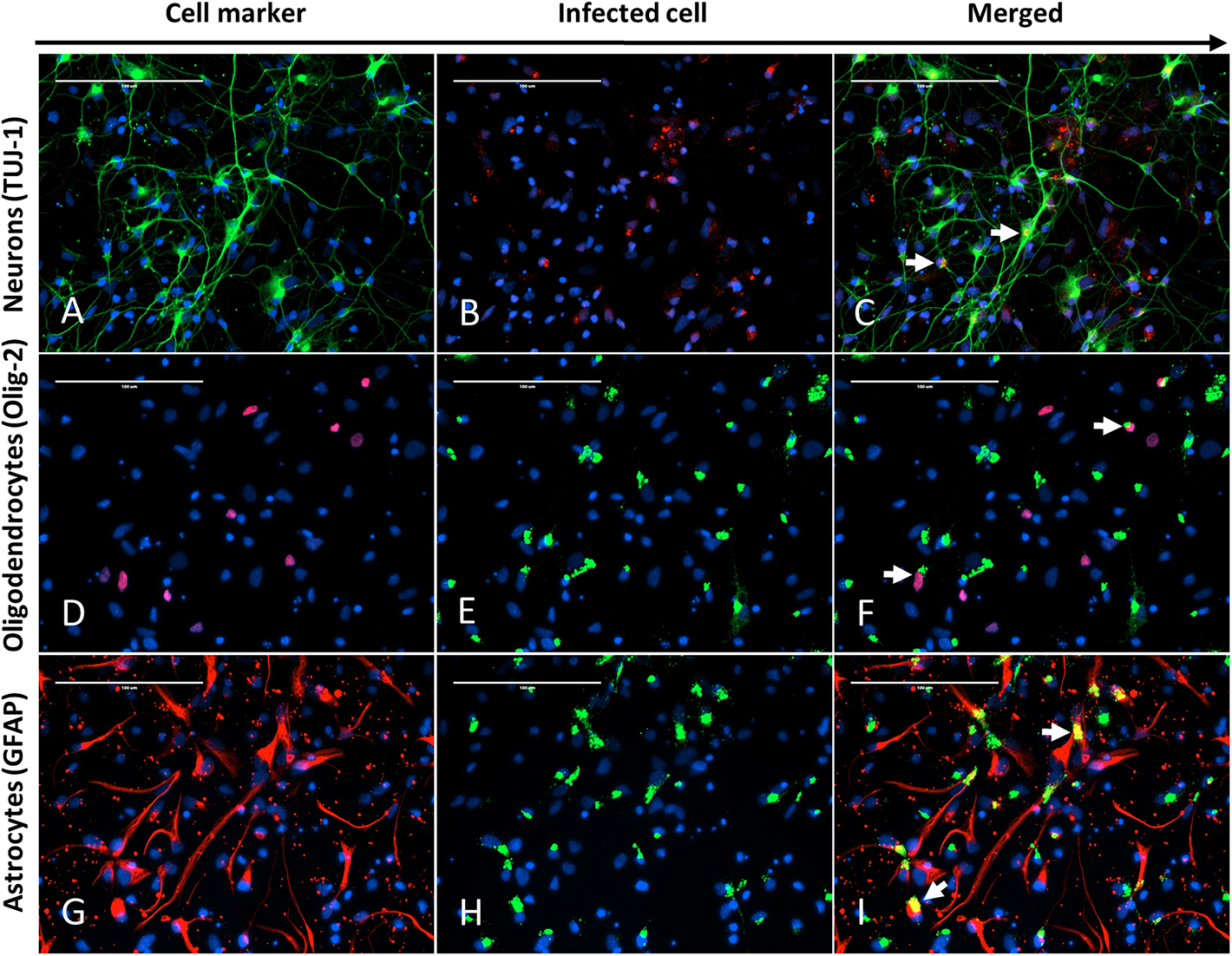
Representative fields of chicken neural cells infected with NDV TxGB strain, 12 hpi (MOI = 10). The same field (400×) was captured for each row and DAPI (pseudo-colored blue) is used for nuclear staining in all images. The first column of each row shows the cells stained for the respective cellular marker: **a** Tuj-1 for neurons (pseudo-colored green), **d** Olig-2 for oligodendrocytes (pseudo-colored red), and **g** GFAP for astrocytes (pseudo-colored red). The second column of each row shows the immunoreactivity for NDV in the same filed: **b** NDV-AP (pseudo-colored red), **e** NDV-BM (pseudo-colored green), and **h** NDV-BM (pseudo-colored green). The third column of each row (**c, f**, and **i**) shows the merged images from the first and second columns to demonstrate the colocalization of NDV and the respective cellular markers. **c** Double IFA signal shows cytoplasmic colocalization of the Tuj-1 and NDV fluorescent signals in scattered cells (white arrows). **f** Double IFA signal shows nuclear (Olig-2) and cytoplasmic (NDV-BM) colocalization of the fluorescent signal in scattered cells (white arrows). **i** Double IFA signal shows cytoplasmic colocalization of the GFAP and NDV fluorescent signals in scattered cells (white arrows)

Primary neural cell cultures were successfully derived. At 24 h after preparation, under bright field microscopy, the population of neural cells was morphologically mixed, composed of stellate and spindle cells (presumptive neurons and astrocytes) and rounder cells with ill-defined morphology. Immunocytochemistry revealed that the mixed population of cells was composed of TUJ-1-, GFAP- and OLIG-2-positive cells consistent with neurons, astrocytes and oligodendrocytes, respectively (Fig. 1a, d, and g). TUJ-1 immunoreactivity highlighted multiple long, fine cytoplasmic extensions, which are consistent with axons and dendrites (Fig. 1a). OLIG-2-reactive cells displayed intranuclear immunofluorescence, consistent with the antibody’s target as a transcription factor (Fig. 1d). GFAP-positive cells had abundant cytoplasm and a more unipolar to bipolar morphology (Fig. 1g).

### Assessing viral replication

Neural cells were infected with LaSota and TxGB NDV strains at multiplicity of infection (MOI) = 10, and supernatants were collected at 1- and 24-h post infection (hpi) and assayed for virus titration. Titers are expressed as EID50/ml and the fold change between 24 hpi and 1 hpi was compared between the viral strains. The mean fold change in EID50/ml of virus in supernatant was significantly higher between 1 hpi and 24 hpi (*P* = 0.015) for TxGB (475-fold increase, 95% CI: 142–810) than for LaSota (53.9-fold increase, 95% CI: − 171–279).

### In vitro NDV infection

**Fig. 2:**
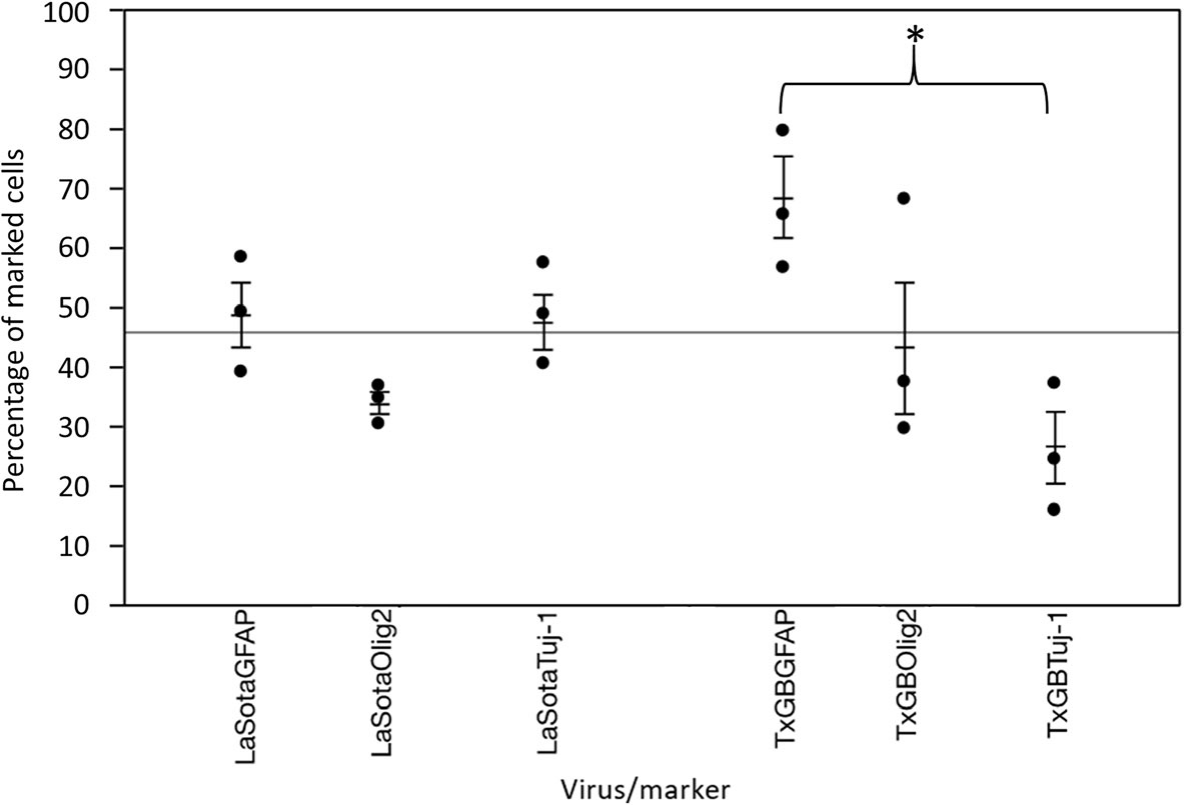
Differential count of NDV^+^/GFAP^+^-, NDV^+^/OLIG-2^+^-, and NDV^+^/TUJ-1^+^-double positive neural cells (In vitro). Data are expressed as the weighted percentage of double-positive cells over the number of cells positive for each cellular marker. *Indicates significant difference between groups (2-way ANOVA with Tukey HSD post-hoc test for multiple comparisons, *p* < 0.05). Bars represent one standard error of the mean and experiment-wide mean is shown as solid line

To determine in vitro cellular tropism of NDV, double-stained (NDV + cell marker) cells were counted after infection with LaSota (non-virulent) or TxGB (virulent, neurotropic) NDV strains, at 1 and 12 hpi. Immunocytochemistry for NDV at 1 hpi was negative in all observed wells (data not shown). While cytopathic effect was not observed in the neural cells at 12 hpi, IFA for NDV demonstrated finely to coarsely, intracytoplasmic, often perinuclear, immunoreactivity, regardless of strain (Fig. 1b [TxGB], 1E [LaSota], and 1H [LaSota]). Overall, there was no difference (weighted [by number of cells] 2-way ANOVA; alpha = 0.05) in percent of cells infected by LaSota or TxGB with 45% (39–51%; 95% confidence interval) and 45% (30–61%; 95% confidence interval) of the total cells in the wells being immunopositive for NDV LaSota or TxGB at 12 hpi. Double immunofluorescence for NDV nucleoprotein (NP) and TUJ-1 or GFAP signals was detected, in merged pictures, as a yellow granular intracytoplasmic fluorescence, as both stain the cytoplasm (Fig. 1c and i). Since OLIG-2 resulted in intranuclear staining and NDV antigen was detected intracytoplasmically, double staining between NDV NP and OLIG-2 signal was defined as NDV NP immunoreactivity adjacent to the Olig-2 positive nuclei (Fig. 1f). The percentage of marker-positive cells that were also NDV positive was counted and compared between viruses and cell types (*n* = 3 wells). At least 20% of all cell types were infected with NDV, regardless of strain (Fig. 2). The weighted percentages were analyzed by a two-way ANOVA with Tukey’s HSD post-hoc multiple comparison test, which demonstrated only one significant difference: TxGB / GFAP vs. TxGB / TUJ1 (*P* < .01) (Fig. 2). Control mock-infected neural cells did not show any fluorescence for NDV NP (Negative controls for each of the cell marker and NDV antigen used in vitro study are included as Additional file 1: Figure S1).

### In vivo NDV infection

**Fig. 3:**
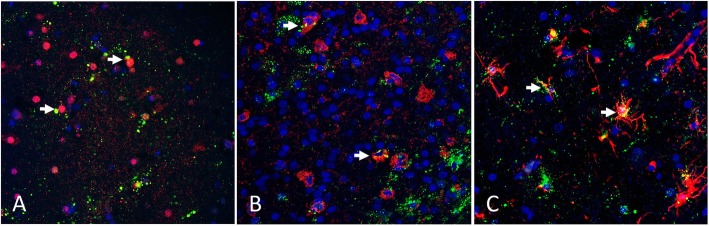
Representative fields of chicken brains infected with NDV **a**: infected with NDV SA60, **b**: infected with NDV TxGB, **c**: infected with NDV Tx450) and co-labeled for cellular markers. In all sections, NDV immunostaining is green and all sections were counterstained with DAPI (blue) to highlight nuclei. **a** IFA for Neu-N (pseudo-colored red). Infected neurons show perinuclear and granular immunofluorescence (pseudo-colored green) adjacent to Neu-N positive nuclei. **b** Fluorescent lectin histochemistry for RCA-I reactivity (pseudo-colored red). Infected microglia demonstrate co-localization of the fluorescent signal (white arrow). **c** IFA for GFAP (pseudo-colored red). Infected astrocytes demonstrate co-localization of the fluorescent signal (white arrow) in the cytoplasmic projections of GFAP-positive cells

Formalin-fixed, paraffin-embedded tissues from a previous study [13] were used to evaluate cellular tropism in vivo. All three vNDV strains (SA60, TxGB, and Tx450) resulted in granular, intracytoplasmic NDV immunoreactivity in scattered cells of the neuroparenchyma, similar to the distribution detected using chromogen based IHC [13]. Merging of the cell marker detection channel with the NDV detection channel demonstrated that all three viruses were detected in neurons, astrocytes, and RCA-I positive cells (cells with microglial and endothelial morphology). Representative images (Fig. 3) demonstrate NDV immunoreactivity in NeuN-positive cells, (Fig. 3a; SA-60 inoculation), RCA-I-positive cells (Fig. 3b; TxGB inoculation), and in GFAP-positive cells (Fig. 3c; Tx450 inoculation). The intracytoplasmic NDV immunostaining within NeuN-positive cells formed large and small, well-defined aggregates. In GFAP-positive cells, the NDV immunoreactivity extended into the stellate GFAP-positive cytoplasmic projections, and frequently surrounded vessels. NDV immunoreactivity was identified in small round to oval RCA-I reactive cells (consistent with microglia) and in RCA-I positive cells that lined vessels (consistent with endothelium). Non-specific binding of anti-NDV antibodies was not observed when DIFA was performed on the brain tissues collected from birds in which intracranial inoculation was performed with only allantoic fluid (negative control group, Additional file 2: Figure S2) [13].

## Discussion

The present study was conducted to 1) define the CNS cell types infected by NDV using double immunolabeling in vitro and in vivo and 2) assess the ability of multiple NDV strains, including velogenic viscerotropic, velogenic neurotropic, mesogenic, and lentogenic (non-virulent) pathotypes, to differentially infect neural cells. In vitro results showed that both velogenic and lentogenic strains could infect cultured neurons, astrocytes, and oligodendrocytes (microglia were untested), in that both viruses had detectable NDV nucleoprotein. Interestingly, there were no differences between viral strain and NP levels in any one cell type.

In vitro, a higher percentage of cultured GFAP-positive cells were also TxGB-NP positive, as compared to TUJ-1-positive cells. However, the in vivo results for TxGB failed to support this difference. Thus, the relevance of this difference in cell culture is not fully determined. Furthermore, even though trypsin, which is required for cleavage of the LaSota F protein [2, 17], was not added to the cell culture, the LaSota strain was able to enter and produce NP in all three tested cell types. This is perhaps due to; 1) the fact that virus source, allantoic fluid, have trypsin-like proteases in the fluid and are ready to infect 2) or the high MOI used for the infections, which may have overwhelmed normal antiviral mechanisms of neural cells. This could also be due the fact that the LaSota was allantoic fluid origin and already had The LaSota strain was not tested in vivo as it is already known that even with intracerebral infection, the LaSota strain lacks the ability to invade the neuroparenchyma [12].

Not only could NDV infect cultured neurons, astrocytes, and oligodendrocytes, but these mixed cultures were permissive for replication of TxGB and LaSota, as demonstrated by the increase in viral titers over time after infection. However, it cannot be determined if all cell types, or only some cell types, are permissive to TxGB replication since all experiments were conducted in mixed cultures. These findings are in contrast to results by Kim and colleagues, who described the inability of LaSota to grow in primary neuronal cells (MOI = 0.1), even after addition of allantoic fluid to allow cleavage of the F protein [18]. It can be speculated that slight changes in the protocol are responsible for this discrepancy. For example, in the experiments herein, viruses were applied with a high MOI_EID50_ (=10) and immunostaining was performed at 12 hpi, whereas Kim and colleagues used a low MOI_pfu_ (= 0.01) and stained cells 48 hpi [18]. Collectively, these results demonstrate that these mixed neural cells are permissive to TxGB and LaSota infection.

Even though the mixed neural cells were infected with a high MOI (=10), 20–80% of any given cell type did not become infected at 12 hpi (i.e., marker-positive, NDV-negative cells), suggesting that neurons, astrocytes, and oligodendrocytes constitute a mixed population with variable permissibility to NDV infection. This may reflect the variability of cellular subtypes that are present in the brain of developing embryos, and possibly the degree of cellular differentiation. The degree of cellular differentiation has been shown to determine permissibility to virus replication, as shown for human hepatitis C virus and varicella-zoster virus in early hepatocyte [19] and neural precursors [20].

In previous animal studies in which TxGB was inoculated via eyelid instillation or subdural, TxGB replicated efficiently in cells within the neuroparenchyma, reflecting its ability to replicate in the brain in live animals [9, 10, 13]. In the work presented here, the in vitro results supported the ability of TxGB to actively replicate in neurons, astrocytes, and oligodendrocytes. Furthermore, the in vivo studies demonstrated that two velogenic strains and one mesogenic strain can infect GFAP-positive cells (astrocytes) and RCA-I-positive cells (microglia and endothelium), as well as the previously reported neurons. The dual reactivity of RCA-I for microglia and endothelium cells was expected [21, 22]. This poses a challenge for definitively identifying microglial infection. Future studies using newer, more specific microglial markers (e.g., IBA-1) would help define the pathogenesis more precisely. It is important to note that the in vivo studies are not able to isolate infectious virions from antigenically defined cells; thus, permissibility of the cell types was not assayed in vivo. In addition, it is important to note that while virus was colocalized in the cell types, an active infection of these cell types was not established. For example, it is possible that microglia cell phagocytosed viral antigen.

The identification that various strains and pathotypes of NDV can infect astrocytes and microglial cells in vivo is consistent with other paramyxoviral infections [15] and suggests that these infected glial cells (astrocytes, oligodendrocytes, and microglia) may contribute to the neuropathogenesis of NDV. Overall, the above data confirm that NDV can infect neuroparenchymal cells other than neurons, and suggest that astrocytes, microglia, and oligodendrocytes may play a role in the neuropathogenesis of virulent NDV by supporting NDV replication and possible spread within the neuroparenchyma of infected chickens.

## Conclusion

This study is the first to use double immunolabeling to determine that a virulent and non-virulent NDV strain are able to simultaneously infect neurons, astrocytes and oligodendrocytes in vitro with an indistinguishable percentage of cells infected between the two viral strains. These data suggest that factors other than cellular tropism are likely the major determinant of the neurotropic phenotype. Additionally, the double immunolabeling was used for the first time to confirm that velogenic and mesogenic strains of NDV can replicate in neuroparenchymal cells other than neurons in vivo, including astrocytes and microglia. This study describes in vitro and in vivo techniques that can be used to further dissect the mechanisms behind the varying pathology of the different NDV pathotypes on the CNS.

## Methods

### Viruses, antibodies, and lectins

Four NDV strains, from repository of the Southeast Poultry Research Laboratory (SEPRL), USDA-ARS, Athens, Georgia, USA, were used: a VVNDV strain (South Africa/08100426/2008 [SA60], ICPI: 1.91 [23]), a VNNDV strain (US/GB/48 [TxGB], ICPI: 1.8 [2, 10]), a mesogenic strain (US/TX4156/2005 [TX450], ICPI: 1.35 [24]), and a non-virulent strain (LaSota, ICPI: 0.00 [2, 10]). Virus stocks were propagated in specific-pathogen-free, 9- to 10-day-old embryonated chicken eggs. Virus titers were determined by limiting dilution in eggs and expressed as embryo infectious dose 50% (EID50) [2]. The EID50 value was used to calculate the multiplicity of infection (MOI) for in vitro cell infection and for confirming viral growth in neural cells. Details on primary antibodies used in this study to detect different subsets of neural cells for double immunofluorescence (DIFA), are reported in Table 1. Briefly, TUJ-1 and NeuN for neurons, GFAP for astrocytes and Olig-2 were used for oligodendrocytes.

**Table 1:** Primary antibodies used on cell cultures and tissue sections

Antibody / Cell	Dilution / Isotype	Raised / Clonality / Cell localization
GFAP / Astrocytes^a^ (Abcam #16997)	1:200 / IgG	Rabbit / Polyclonal / Cytoplasmic
GFAP / Astrocytes^b^ (MU020-UC)	1:200 / IgG1	Mouse / Monoclonal / Cytoplasmic
TUJ-1 / Neurons^a^ (Abcam #14545)	1:1000 / IgG2a	Mouse / Monoclonal / Cytoplasmic
Neu-N / Neurons^b^ (MAB377)	1:400/ IgG1	Mouse /Monoclonal / Nuclear
OLIG-2 / Oligodendrocytes^a^ (Genetex #62440)	1:250 / IgG	Rabbit / Monoclonal / Nuclear
Biotinylated *Ricinus communis* Agglutinin-1 (RCA-1)^b^ (B-1085)	1:500 / Biotinylated Lectin	Cytoplasmic
Anti-NDV-NP^a^	1:1000 / IgG	Rabbit / Polyclonal / Cytoplasmic
NDV-BM (Novus Biologicals #NBP2–11633)	1:2000 / RNP*	Mouse / Monoclonal /Cytoplasmic
Anti-NDV-NP^b^	1:800 / IgG	Rabbit / Polyclonal / Cytoplasmic

^a^for double immunofluorescent assay (in vitro)

^b^for double immunofluorescent assay (in vivo)

*RNP; Ribonucleoprotein

### Source of eggs, tissues, and egg-incubation conditions

Fertilized, specific-pathogen-free (SPF), White Leghorn chicken eggs were obtained from the Southeast Poultry Research Laboratory (Athens, GA, USA) SPF White Leghorn flock. Eggs were incubated at 37 °C and 50% relative humidity in a standard egg incubator for 11 days, corresponding to embryo stage 37 [25]. Eggs were candled daily to assess embryo viability; non-viable embryos were discarded. At day 11 of incubation embryos were euthanized by placing eggs at 4 °C for 30 min in a cold room. After this period, eggs were decontaminated by lightly spraying with 70% ethanol (in distilled water) and then transferred to a biosafety cabinet to harvest neural cells. For the in vivo component of the study, archived [13] formalin-fixed paraffin-embedded brains from day-old SPF chicks (in the original experiment day old White Leghorn Chicks were obtained from the Southeast Poultry Research Laboratory [Athens, GA, USA] SPF White Leghorn flock and were euthanized by cervical dislocation) infected with three vNDV strains were used for double immunofluorescence (see below).

### Preparation of primary chicken neural cells

Harvest and manipulation of neural cells was conducted in a laminar flow biosafety cabinet under sterile conditions. For each preparation, 8–10 embryos were used. Neural cell preparation was carried out in a similar as described by Crump and colleagues, with slight modifications [26]. Briefly, the cerebral hemispheres of the embryos were removed and immediately submerged in ice-cold phosphate-buffered saline (PBS^−−^, Ca^2+^ and Mg^2+^ free, HyClone®). After peeling off the meninges and choroid plexuses using a stereomicroscope, the cerebral hemispheres were minced using a scalpel blade and then transferred into tubes containing 2 ml of trypsin-EDTA 0.25% (Gibco®, #25200–056). The mixture was gently pipetted up and down and then incubated for 15 min at 37 °C in a water bath, gently swirling every 5 min to facilitate tissue dissociation. Trypsin was neutralized by adding 2 ml (1:1 ratio) of Neurobasal medium (NBM), which was the culture medium used in this study to maintain neural cells [composition: Neurobasal™ medium (Gibco®, #21103–049) with 2% B27 serum-free supplement (Gibco®, #17504–044), 1% pen/strep (Gibco®, #15070–063), and 1% L-glutamine (Gibco®, #25030–081)]. After centrifugation (4 min at 1400 rpm), the supernatant was discarded, and the cell pellet was resuspended in 5 ml of NBM, passed through a cell strainer (40-μm nylon, BD Falcon, #352340), and then pre-plated for 1 h in a 100-mm uncoated cell culture dish containing 8 ml of NBM. Pre-plating was included as an additional step to decrease contamination with red blood cells (RBC) and fibroblasts. Following incubation, the cell suspension was aspirated from the dish, centrifuged (as previously), resuspended in NBM, and finally plated with NBM in wells pre-coated with poly-D-lysine (Sigma, #P6407-5MG), to increase cellular adherence. Cells were plated at a density of 2 × 10^5^ cells/well in culture slides (BD Falcon, #354104) or 3 × 10^5^/well in 24-well plates. Neural cells were cultured at standard conditions (37 °C with 5% CO_2_) for a maximum of 48 h before any assay was carried out.

### Infection of primary mixed neural cells with NDV strains

Primary mixed neural cells were prepared in 24-well plates at a concentration of 3 × 10^5^ / well. For LaSota, TxGB and control groups, three wells (one for each double stain, see next section) were plated from three independently collected batches of neural cells (prepared from different groups of embryos), which constituted three biological replicates. At 24 h post-plating, cells were infected with NDV strain LaSota or TxGB at a multiplicity of infection (MOI) = 10. At the time of infection, in order to calculate the MOI, cells from three extra wells were washed with PBS^−−^, dissociated with trypsin, and counted with an automatic cell counter (Nexcelom Bioscience Cellometer Auto T4). Infection was carried out as follows: NBM was discarded from each well and cells were covered with 400 μl/well of a suspension of virus in NBM (MOI = 10) for 1 h at 37 °C, gently rocking the plates every 15 min in order to optimize virus spread across the surface of the wells. After virus absorption, cells were washed three times with PBS^−−^ and then cultured with NBM. For mock-infected cells (control), simple NBM without virus was added to the wells, leaving the other passages the same as described. At 1 (right after the first wash), 12, and 24 h post-infection (hpi), 200 μl of supernatant were harvested for titration, and then cells were fixed in 4% paraformaldehyde for DIFA (see below).

### Assessing viral replication

The magnitude of viral replication in the supernatant was assessed by titration of the amount of virus using limiting dilutions in eggs (titers expressed as EID50 units) [6]. The fold changes between the titer produced after 24 h and the titer produced after 1 h in cell culture were statistically compared between viral strains using a t test (alpha = .05) (JMP Pro 13.0.0, Cary, NC).

### Double immunofluorescence assay for neural cell characterization and NDV detection

Double immunofluorescence assay was used to characterize the type of cells in the mixture of primary neural cells, as well as to determine NDV infection in these cells. Neurons, astrocytes, and oligodendrocytes were immunostained with specific antibodies (see Table 1).

In order to facilitate double staining with the other cellular markers to detect NDV, two antibodies isotypes [one raised in rabbit and one in mice] against NDV NP were used. The list of primary and secondary antibodies, as well as their specifications and working concentrations is presented in Table 1 and Table 2, respectively. DIFA was conducted in 24-well plates and the protocol was the same for all antibodies used in this research, as outlined below.

**Table 2:** Combinations of primary and secondary antibodies used on cell cultures and tissue sections

Primary antibody for cell marker	Secondary (wavelength absorbance in nm)	Primary antibody for NDV infection	Secondary (wavelength absorbance in nm)
GFAP^a^	Red (594) goat anti-rabbit	NDV-BM	Green (488) goat anti-mouse
TUJ-1^a^	Green (488) goat anti-mouse	NDV-AP	Red (594) goat anti-rabbit
OLIG-2^a^	Red (594) goat anti-rabbit	NDV-BM	Green (488) goat anti-mouse
GFAP^b^	Red (555) goat anti-mouse	NDV NP synthetic peptide	Green (488) goat anti-rabbit
NeuN^b^	Red (555) goat anti-mouse	NDV-NP synthetic peptide	Green (488) goat anti-rabbit
RCA-1^b^	Red (555) Biotin binding streptavidin	NDV-NP synthetic peptide	Green (488) goat anti-rabbit

^a^antibodies used in the DIFA for chicken neural cells (in vitro) infected with LaSota and TxGB NDV strains

^b^antibodies used in the DIFA for chicken brains (in vivo) infected with SA60, TxGB and Tx450 NDV strains

DIFA was performed in order to assess infection of low (LaSota wild type) and high (Texas GB) virulence NDV strains in different neural cell populations. Briefly, supernatant was removed and used for virus titration from each well, and cells were washed with PBS^++^ (supplemented with Ca^2+^ and Mg^2+^, HyClone®), and fixed with 4% paraformaldehyde for 15 min at room temperature. After fixation, cells were washed three times with PBS^++^, permeabilized and blocked with a PBS^−−^ solution containing 4% normal goat serum, 0.1% Triton X-100 and 1% polyvinylpyrrolidone for 45 min, washed twice with high salt buffer (HSB; 0.05 M Tris base, 0.25 M NaCl), and then incubated with the primary antibodies diluted in blocking buffer (300 μl/well) at room temperature for 1 h. The primary and secondary antibody combinations are reported in Table 2. After incubation, cells were washed three times for 5 min with wash buffer (HSB containing 5% Tween 20), once with HSB, and finally incubated with fluorophore-conjugated secondary antibodies (300 μl/well) at room temperature in dark chamber for 1 h. After incubation, cells were washed three times (5 min each) with wash buffer, once with HSB, covered with Prolong® Gold containing DAPI (Molecular Probes, #P36935), and then coverslipped. Cells were observed and images were captured using the EVOS® FL microscope (Life Technologies). Uninoculated cells were used to control for the specificity of the anti-NDV antibodies.

Two 400× fields for each biological replicate at 12 hpi were photographed under fluorescence (EVOS® FL microscope, Life Technologies) for GFP (peak absorption 488 nm), Texas Red (594 nm) and DAPI (345 nm). Images for each fluorescent spectrum were merged and saved. Images were then analyzed by manually counting the number of cells for NDV NP and cellular markers (Image-Pro Express 6.0), as well as the number of cells that were not double stained. For each well (i.e., two fields of each NDV/marker double staining), 110–201 cells were counted. For each NDV strain (LaSota or TxGB), the number of double-stained cells was statistically analyzed with two-way ANOVA followed by the Tukey HSD post-hoc multiple comparison test (JMP Pro 13.0.0, Cary, NC).

### Fluorescent immuno- and lectin histochemistry

To determine if NDV infects a subset of glial cells (astrocytes and microglia) in vivo, archived samples from a previous study were used [13]. The secondary antibody for Olig-2 was the same isotype as the NDV antibody (goat anti-rabbit). As the best primary antibody for sensitive NDV detection is the rabbit polyclonal version, which conflicts with the olig-2 markers that worked in the in vitro study. Therefore, due to lack of additional markers for in vivo oligodendrocyte double-labeling data were not included in the paper. Briefly, day-old White Leghorn chickens had been inoculated intracranially with 10^4^ 50% tissue culture infectious dose (TCID_50_) units of three NDV strains. These included TxGB, which was used for the in vitro experiments, SA60 (VVNDV) and Tx450 (mesogenic). The LaSota strain was not used as it has been shown that LaSota virus does not penetrate the neuroparenchyma. Euthanasia was scheduled at specific time points and birds with significant clinical signs had also been euthanized. Immediately after euthanasia, the head from each bird was harvested and fixed in neutral-buffered 10% formalin for 48 h. Three brains from each inoculation group were randomly selected. Samples were collected at 2 days post infection (dpi) for birds infected with velogenic strains (SA60 and TxGB). As these virulent viruses cause death of all birds by 2 dpi therefore, later time points (4 dpi) was not available from these two group of birds. The mesogenic NDV strain (Tx450) did not cause mortality in the infected birds therefore brain tissues were collected at 4 dpi. After decalcification in Kristensen’s solution for 20 min, samples were then transferred to 70% ethanol. Brain sections were routinely processed and embedded in paraffin, and sagittal, 3-μm-thick sections were cut for fluorescent IHC staining.

Dual immunofluorescence assay was used to co-label NDV and cellular markers. Unstained paraffin sections were deparaffinized in Hemo-De (Fisher Scientific, Pittsburgh, PA) and subjected to antigen retrieval by autoclaving for 10 min at 121 °C in 1x unmasking solution (Vector Laboratories, Burlingame, CA). Sections were blocked by incubation for 20 min with blocking solution (2% fetal bovine serum, 1% goat serum in 1× PBS^−−^) at room temperature in a humidified chamber. Sections were then rinsed twice for 3 min each and incubated for 2 h with a cocktail of primary antibodies (Table 1) in blocking solution. Unbound primary antibodies / lectins were removed with three washes in 1× PBS^−−^ for 5 min each. Bound primary antibodies were then detected by incubating tissue sections in a cocktail of appropriate secondary antibodies depending on the primary antibody pairing (Goat anti-rabbit IgG (H + L), Alexa 488, Goat anti-mouse IgG (H + L) Alexa 555) (Table 2).

Bound lectin was detected through application of streptavidin-conjugated fluorophore Alexa 555. All fluorophore-conjugated reagents were diluted 1:400 with blocking solution and applied for 1 h in a humidified chamber at room temperature and protected from direct light. Tissue sections were washed with PBS^−−^ for 2 × 10 min and with water 5 min. Slides were immersed in 10 mM CuSO4.5H_2_O, pH 5.0 for 15 min to reduce autofluorescence. After rinsing with water and then PBS, the sections were coverslipped (ProLong® Gold Antifade Mountant with DAPI, Life Technologies, Eugene, OR, USA). Controls included mock-inoculated brains (NDV-negative), as well as sections stained with isotype controls (not available for RCA-I staining) and sections probed without the secondary antibody. From each of the three birds for each group, images from the telencephalon (6 images), thalamus (2 images), midbrain (2 images), and hindbrain (2 images) were acquired with an Olympus TF fluorescent microscope and recorded using Olympus cellSens image analyzing software, version 1.5 (Olympus corporation) and manually analyzed using Image J, version 1.49 (NIH, USA, http://imagej.nih.gov/ij/download.html).

## Additional files

Additional file 1: **Figure S1.** Representative images of primary neural cells infected with only allantoic fluid and then double stained with each of the cell markers and viral antigen. White arrows in merged images (C, F and I) show cells stained with specific cell markers. (TIF 3354 kb)

Additional file 2: **Figure S2**. Representative images of chicken brain tissue infected with only allantoic fluid and then double stained with each of the cell markers and viral antigen. White arrows in merged images (C, F and I) show cells stained with specific cell markers. Yellow arrow in image I, shows non-specific binding of RCA-I to the endothelial cells of blood vessels. (TIF 3371 kb)

## Data Availability

The datasets used and/or analyzed during the current study available from the corresponding author on reasonable request.

## Abbreviations

CNS: Central nervous system
CSF: Cerebrospinal fluid
DAPI: 4′,6-diamidino-2-phenylindole
DIFA: Double immunofluorescence assay
EID50: Embryo Infectious Dose 50%
GFAP: Glial fibrillary acidic protein
GFP: Green fluorescent protein
HPI: Hours post infection
HSB: High salt buffer
ICPI: Intracerebral Pathogenicity Index
IHC: Immunohistochemistry
MOI: Multiplicity of infection
NBM: Neurobasal medium
ND: Newcastle disease
NDV: Newcastle disease virus
NP: Nucleoprotein
PBS: Phosphate buffer saline
RCA-1: *Ricinus communis* agglutinin type. 1
RNP: Ribonuclear protein
SEPRL: Southeast poultry research laboratory
SPF: Specific pathogen free
TCID-50: Tissue culture infectious dose 50%
VNNDV: Velogenic neurotropic NDV
VVNDV: Velogenic viscerotropic NDV
