# Supplementary figures and images for: Tropism of Newcastle disease virus strains for chicken neurons, astrocytes, oligodendrocytes, and microglia
Source: BMC Vet Res. 2019 Sep 4;15:317. doi: 10.1186/s12917-019-2053-z (PMC6727330; doi:10.1186/s12917-019-2053-z)

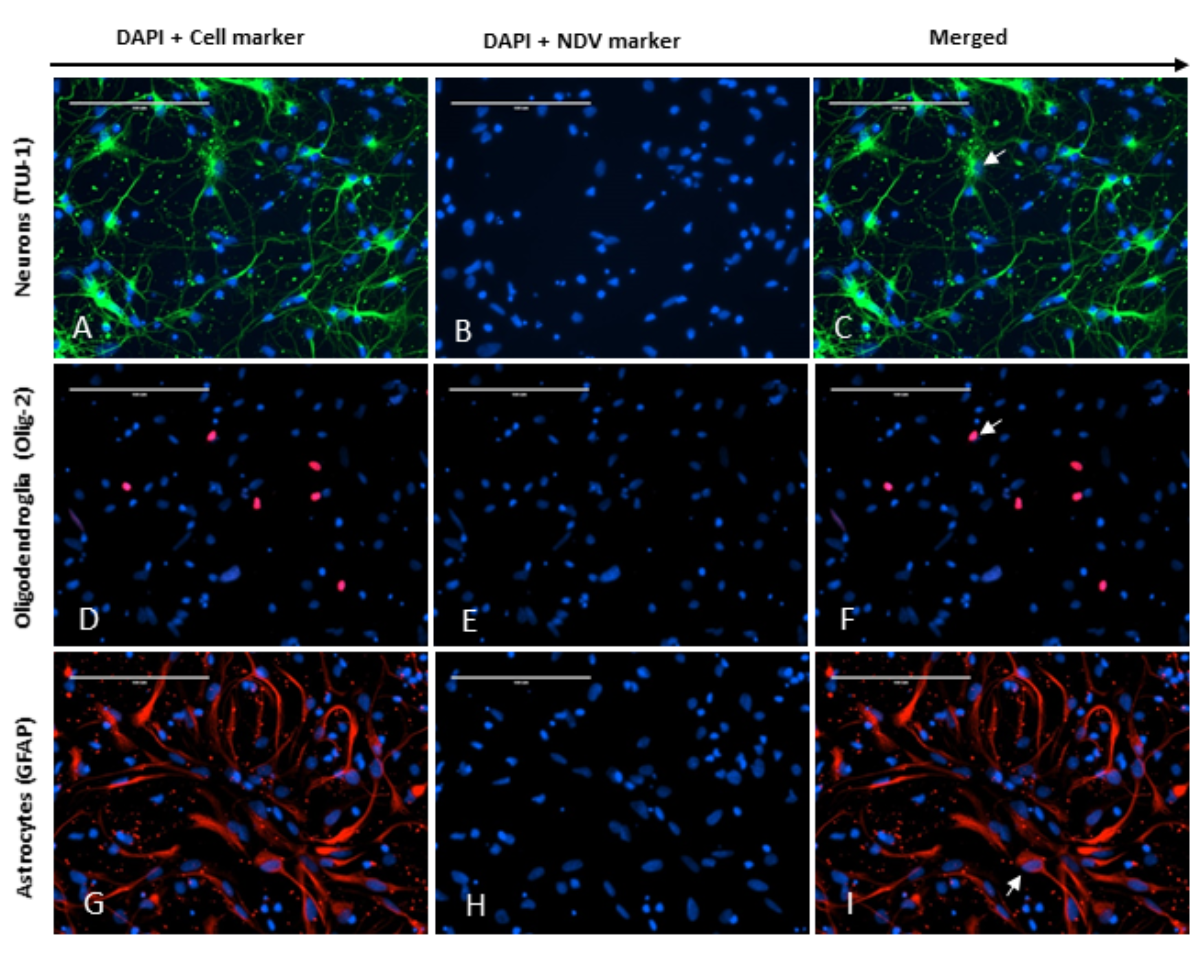

Supplement: Supplementary file 1 — Figure S1. Representative images of primary neural cells infected with only allantoic fluid and then double stained with each of the cell markers and viral antigen. White arrows in merged images (C, F and I) show cells stained with specific cell markers. (TIF 3354 kb) [file 12917_2019_2053_MOESM1_ESM.tif]

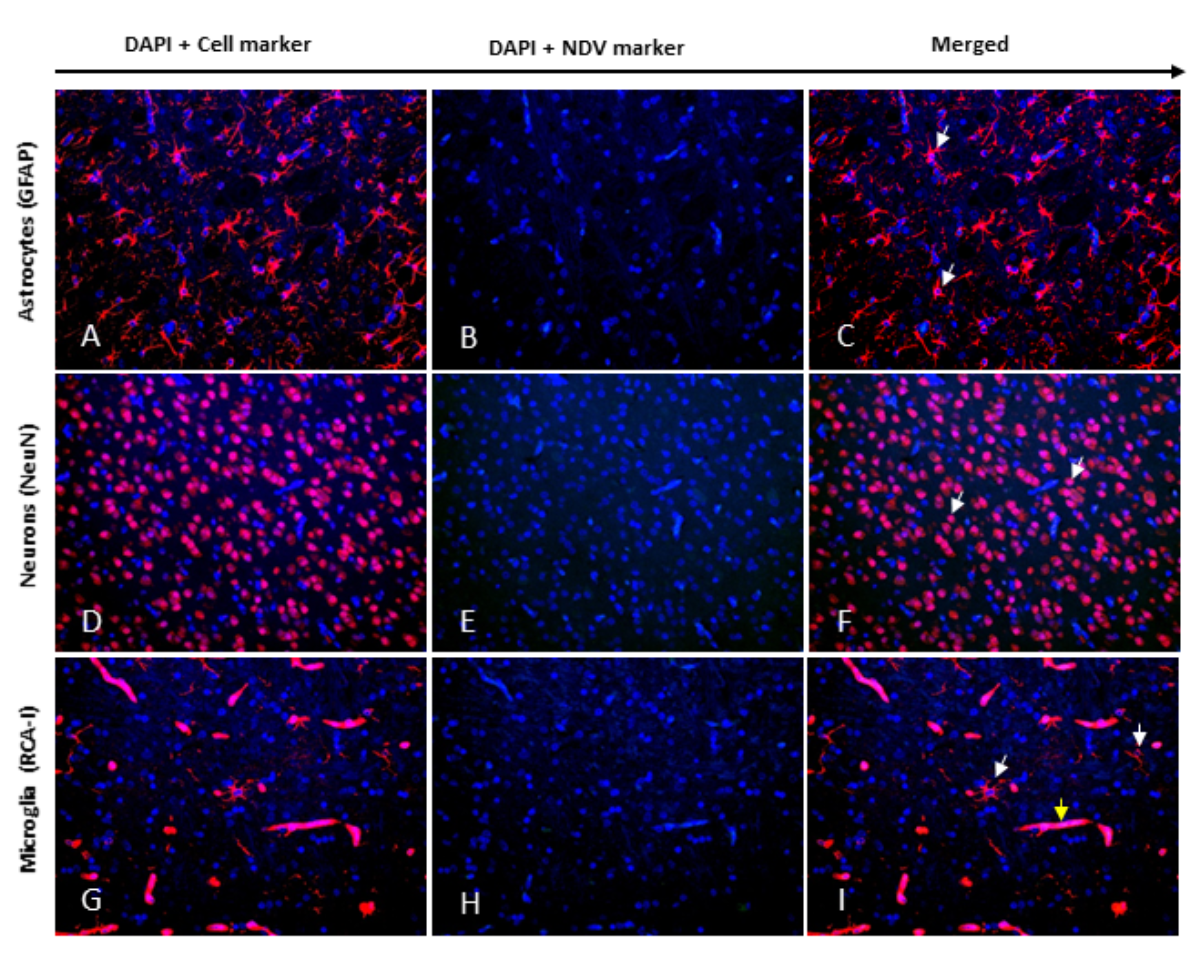

Supplement: Supplementary file 2 — Figure S2. Representative images of chicken brain tissue infected with only allantoic fluid and then double stained with each of the cell markers and viral antigen. White arrows in merged images (C, F and I) show cells stained with specific cell markers. Yellow arrow in image I, shows non-specific binding of RCA-I to the endothelial cells of blood vessels. (TIF 3371 kb) [file 12917_2019_2053_MOESM2_ESM.tif]
